# Prognostic and clinicopathological significance of SNHG6 in human cancers: a meta-analysis

**DOI:** 10.1186/s12885-020-6530-3

**Published:** 2020-01-30

**Authors:** Si Zhao, Hanlong Zhu, Ruonan Jiao, Xueru Wu, Guozhong Ji, Xiuhua Zhang

**Affiliations:** Medical Centre for Digestive Diseases, Second Affiliated Hospital, Nanjing Medical University, Nanjing, Jiangsu Province People’s Republic of China

**Keywords:** Long non-coding RNA, SNHG6, Cancer, Prognosis, Clinical parameters

## Abstract

**Background:**

Recently, accumulating evidence has suggested that the aberrant expression of SNHG6 exists in a variety of tumors and has a correlation with poor clinical outcomes across cancer patients. Considering the inconsistent data among published studies, we aim to assess the prognostic effect of SNHG6 on malignancies.

**Methods:**

We retrieved relevant publications in Web of Science, Embase, MEDLINE, PubMed and Cochrane Library based on predefined selection criteria, up to October 1, 2019. Pooled hazard ratios (HRs) and odds ratios (ORs) with 95% confidence intervals (CIs) were utilized to evaluate the correlation between SNHG6 and overall survival (OS), recurrence-free survival (RFS) and progression-free survival (PFS) as well as clinicopathology.

**Results:**

In total, 999 patients from 14 articles were enrolled in our meta-analysis. The results revealed that augmented SNHG6 expression was significantly correlated with poor OS (HR = 2.20, 95% CI = 1.76–2.75, *P* < 0.001) and RFS (HR = 3.10, 95% CI = 1.90–5.07, P < 0.001), but not with PFS (HR = 2.11, 95% CI = 0.82–5.39, *P* = 0.120). In addition to lung cancer and ovarian cancer, subgroup analysis showed that the prognostic value of SNHG6 across multiple tumors was constant as the tumor type, sample size, and methods of data extraction changed. Moreover, cancer patients with enhanced SNHG6 expression were prone to advanced TNM stage (OR = 3.31, 95% CI = 2.46–4.45, *P* < 0.001), distant metastasis (OR = 4.67, 95% CI = 2.98–7.31, P < 0.001), lymph node metastasis (OR = 2.59, 95% CI = 1.41–4.77, *P* = 0.002) and deep tumor invasion (OR = 3.75, 95% CI = 2.10–6.69, P < 0.001), but not associated with gender, histological grade and tumor size.

**Conclusions:**

SNHG6 may serve as a promising indicator in the prediction of prognosis and clinicopathological features in patients with different kinds of tumors.

## Background

It is widely acknowledged that cancer is a major cause of death with increasing morbidity rates and decreasing survival rates. According to American Cancer Society estimates, the projected numbers of newly diagnosed cases and deaths are 17.6 and 6.0 million, respectively, in the United States in 2019 [1]. Although there is continuous study of the multidisciplinary treatment of cancers by researchers, the prognosis still presents an enormous clinical challenge [2]. Therefore, increasing attention should be paid to identifying innovative and effective biomarkers that play a crucial role in earlier diagnosis and therapeutic decision-making for patients with different kinds of tumors.

In recent years, substantial advancement in the detection of molecular targets has been a catalyst for the prediction of cancer progression and prognosis by various methods including exosomes, cell-free DNAs, non-coding RNAs (ncRNAs), etc., which have garnered widespread interest among researchers. Studies have revealed that ncRNAs are implicated in a myriad of fundamental biological functions and account for 98% of the human genome [3, 4]. Long non-coding RNAs (lncRNAs), which are emerging as an important type of ncRNA, are transcripts greater than 200 nucleotides in length that are incapable of encoding proteins considering their lack of specific open-reading frames (ORFs). For this reason, they are historically speculated as transcriptional noise [5]. Nonetheless, with the in-depth investigation of their mechanisms, mounting evidence has revealed that lncRNAs are involved in numerous physiological and pathological processes, thus affecting transcriptional regulation, cellular scaffold orchestration, protein localization and chromatin modification [6, 7]. In particular, mutation or aberrant expression of lncRNAs are closely related to tumorigenesis, tumor invasion and metastasis [8, 9], which suggests that lncRNAs acting as tumor suppressors or oncogenes have the potential to be important markers for cancer management.

With the development of high-throughput RNA sequencing techniques and bioinformatic algorithms, a spectrum of lncRNAs have been identified. The small nucleolar RNA host gene 6 (SNHG6; also termed U87HG), a subclass of lncRNA molecules, pervasively participates in gene modulation through functioning as an oncogene in different human cancers, including colorectal cancer [10–14], glioma [15], osteosarcoma [16], breast cancer [17], ovarian cancer [18], lung adenocarcinoma [19], oesophageal cancer [20], gastric cancer [21] and liver cancer [22]. Studies have proven that the upregulation of SNHG6 plays multiple critical roles in cell differentiation, proliferation, apoptosis, and multidrug resistance [23, 24]. Currently, despite diverse articles that unveil the link between SNHG6 and cancers, the prognostic value remains contradictory or inconclusive, which may be attributed to limited sample size and methodology. Hence, we integrate pertinent studies to concretely delineate the correlation between SNHG6 and prognosis or clinicopathological features in the form of a meta-analysis of multiple malignancies.

## Methods

### Search strategies

We conducted a comprehensive search, through the Web of Science, Embase, MEDLINE, PubMed and Cochrane Library from inception to October 2019, for available articles that reported the correlation of SNHG6 expression with prognosis and clinicopathological features in human cancers. The key search terms were used as follows with multiple combinations: (“small nucleolar RNA host gene 6” OR “SNHG6” OR “ENSG00000245910”) AND (“carcinoma” OR “cancer” OR “tumor” OR “neoplasm” OR “malignancy”) AND (“prognosis” OR “prognostic”). Further manual inspection was performed to improve the integrity of the eligible papers by going through the title and abstract. Moreover, references in relevant publications were also browsed.

### Inclusion and exclusion criteria

Inclusion criteria: (1) The patients in the original research were definitively diagnosed with cancers by histopathology; (2) The samples were grouped according to the SNHG6 expression level, which was detected by quantitative reverse transcription-polymerase chain reaction (qRT-PCR) or in situ hybridization (ISH); (3) The relationship between the SNHG6 expression and the clinicopathological parameters and at least one pathological feature, such as sex, histological grade, tumor size, TNM stage, lymph node metastasis, distant metastasis or tumor invasion depth, of cancer patients was investigated; (4) The studies described the association of SNHG6 expression with medical outcomes in cancer patients, including OS, PFS and RFS; and (5) HRs and 95% CIs were available to be directly extracted from the article or indirectly calculated by the Kaplans-Meier curves.

Exclusion criteria: (1) Reviews, letters, editorials, case reports, meta-analysis, and conference summaries with non-original data; (2) Insufficient or duplicate data for HR and 95% CI estimation; (3) Studies were carried out on animal specimens.

### Data extraction and quality assessment

Pertinent data extraction was conducted independently by two investigators (SZ and HZ) from identified research in agreement with prescribed standards, during which disagreements were resolved by reaching a consensus on all contents. The extracted data elements mainly included the following information: study ID (lead author plus publication year), region, time of sample collection, cancer type, age, number of patients, endpoints (OS/RFS/PFS), assay method and approximate cut-off value defined for SNHG6 expression levels. Additionally, clinical-pathological parameters, including sex, histological grade, tumor size, TNM stage, lymph node metastasis, distant metastasis and tumor invasion depth, were also extracted. Data were preferentially obtained from multivariate analysis. The quality of retrieved papers was evaluated on the basis of the Newcastle-Ottawa Scale (NOS), in which the scores varied from 0 to 9 [25], and a score greater than 5 was regarded as a high-quality document.

### Statistical analysis

The pooled HR with the corresponding 95% CI was utilized to analyse the association between SNHG6 expression and OS/RFS/PFS in cancer patients. The effect of SNHG6 expression on clinicopathological features was described as the combined odds ratio (OR) and 95% CI. The study was recognized as statistically significant when an observed HR/OR > 1 and 95% CI did not contain 1. Cochran’s Q and Higgins I2 tests were applied to assess heterogeneity across studies. We defined the existence of heterogeneity as I2 > 50% and *P* < 0.10, and then the random-effect framework was used; otherwise, the fixed-effect model was adopted for analysis. Both Begg’s and Egger’s tests were performed to quantitatively reflect potential publication bias. Meanwhile, sensitivity analysis was employed to validate the reliability of the results. In the present meta-analysis, statistical analysis was conducted using STATA 14.0, and *P* < 0.05 indicated that the difference was statistically significant.

## Results

### Literature search and study characteristics

**Fig. 1:**
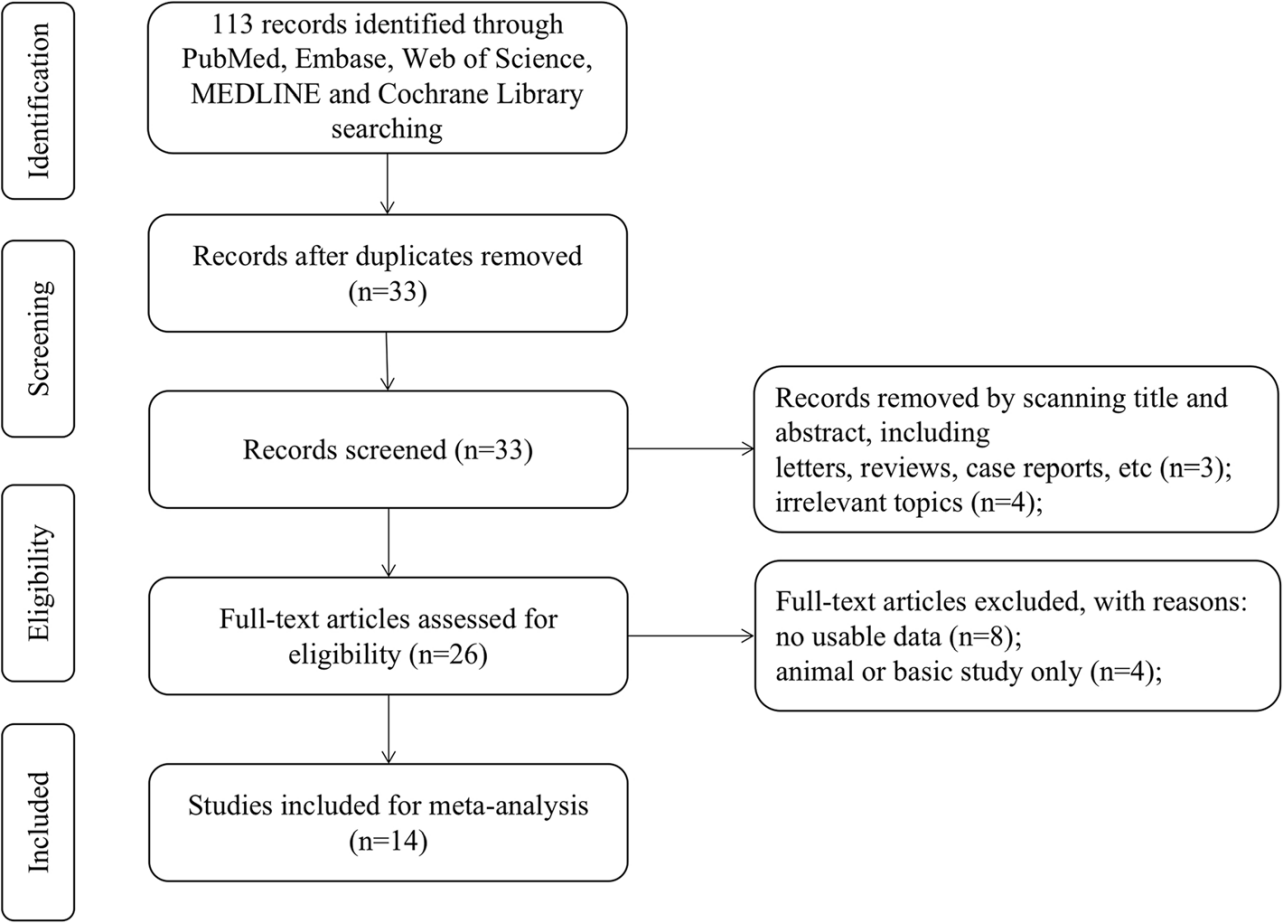
Flow diagram of the study search and selection process

A total of 113 articles were originally retrieved through an electronic database search. Ultimately, 14 articles with a total of 999 patients were considered qualified in light of the defined search strategy. The detailed selection process is shown in Fig. 1. Among the articles, 13 reported the correlation of SNHG6 expression with OS, whereas only two studies reported the correlation between SNHG6 expression and RFS and PFS. As an insufficient number of studies included the PFS and DFS, OS was used as the predominant survival indicator. All of the included studies were performed in China and were published from 2016 to 2019 with a mean sample size of 71. In terms of the type of cancer, 5 studies referred to colorectal cancer [10–14], 2 studies related to glioma [15, 26], 2 studies involved osteosarcoma [16, 27], and the remaining cohorts were on ovarian cancer [18], lung cancer [19], oesophageal cancer [20], gastric cancer [21], and liver cancer [22]. Of the 14 studies included, 6 had survival dates in the original literature, and the survival dates for the others were determined with graphical survival plots following the published method proposed by Tierney et al. [28]. The expression level of SNHG6 was measured in cancerous tissue and detected by qRT-PCR, on the basis of which the patients were divided into high and low SNHG6 groups. The median value was the most commonly utilized for the cut-off values. Due to an average NOS score of 7, our cohort was considered to have better methodological quality. The basic characteristics of the involved studies are presented in Table 1.

**Table 1:**
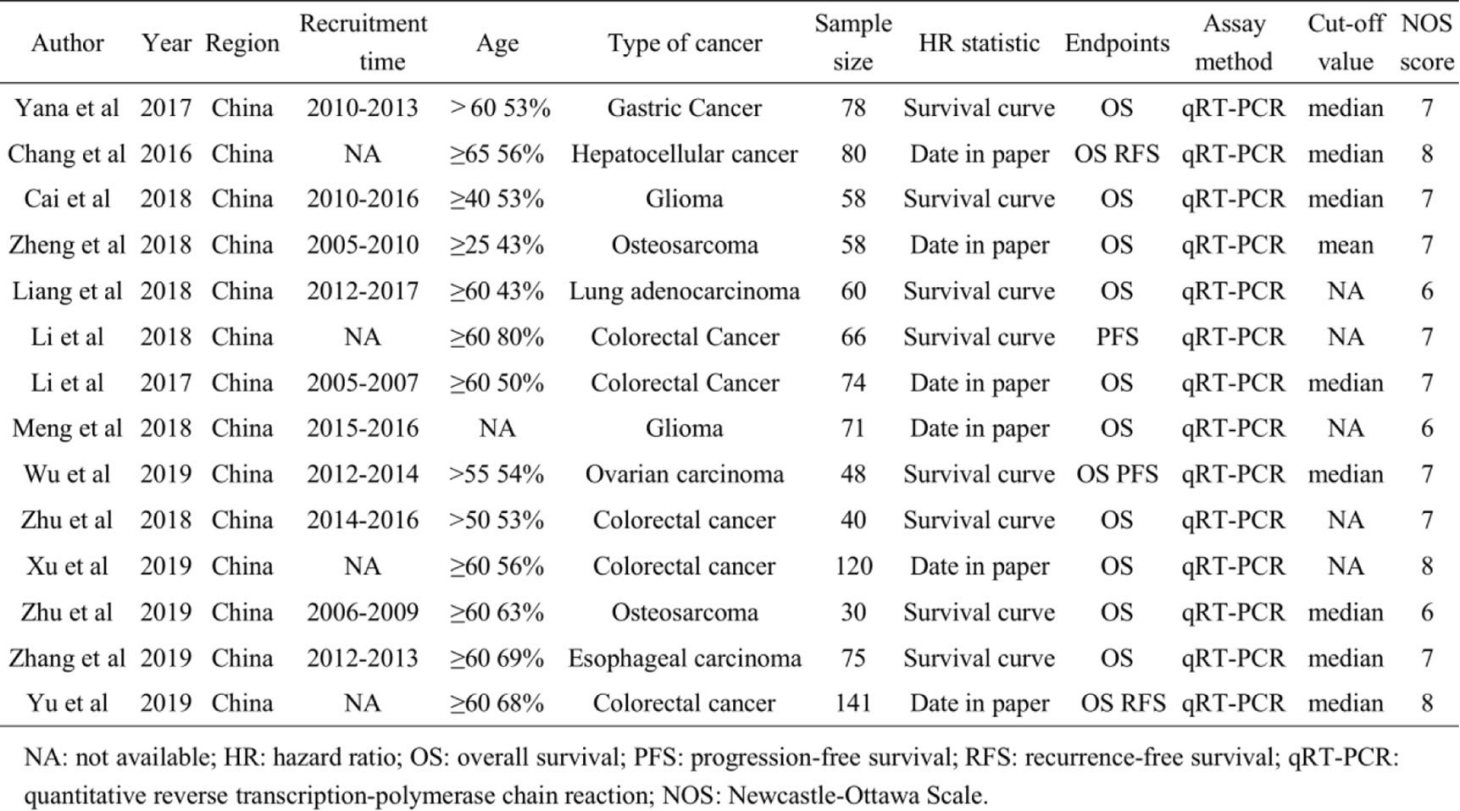
Charactersitics of included studies in this meta-analysis

*NA* not available, *HR* hazard ratio, *OS* overall survival, *PFS* progression-free survival, *RFS* recurrence-free survival, *qRT-PCR* quantitative reverse transcription-polymerase chain reaction, *NOS* Newcastle-Ottawa Scale

### The correlation of SNHG6 expression with clinical-pathological features

#### SNHG6 and TNM stage

**Fig. 2:**
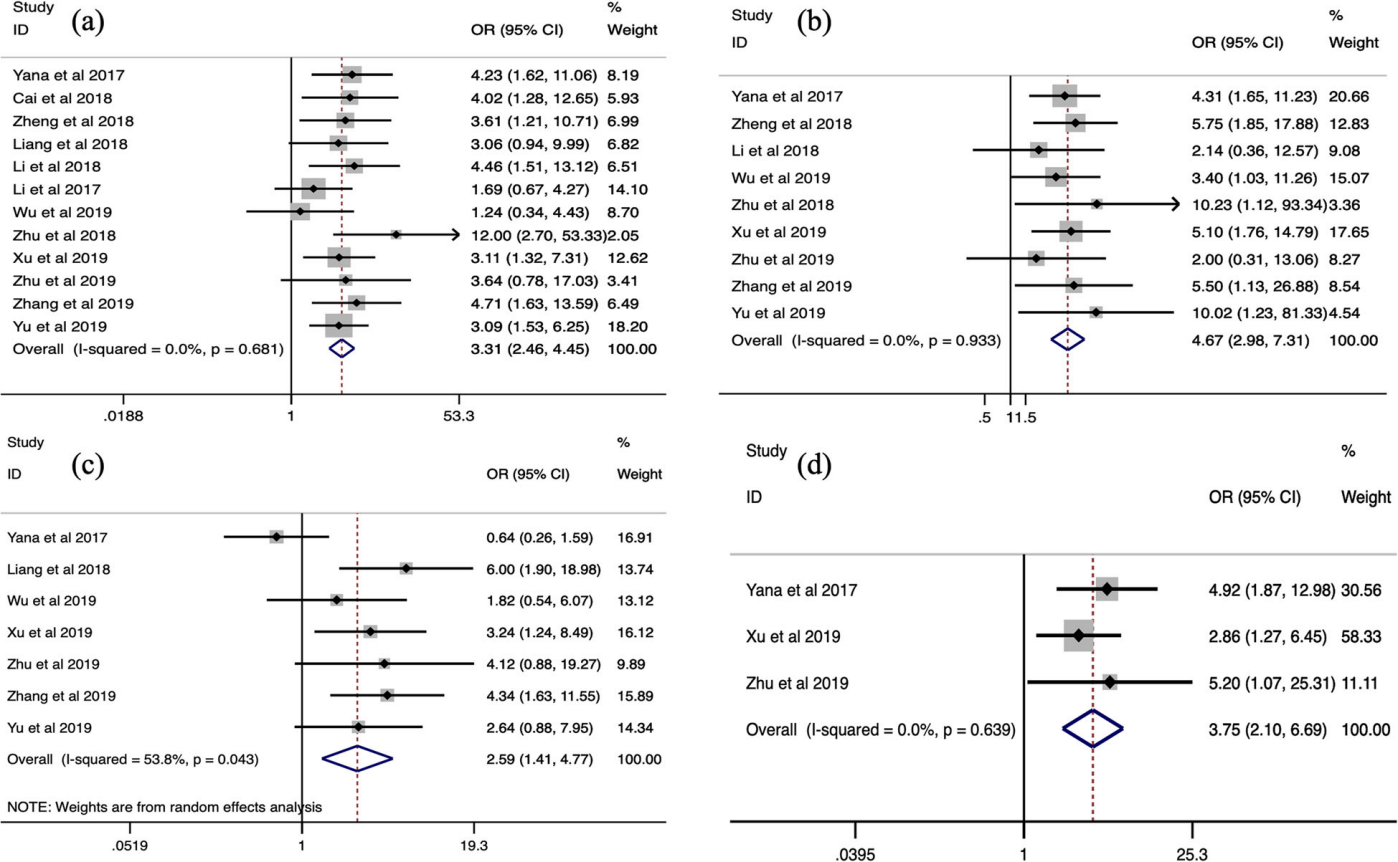
Forest plot for the association between SNHG6 expression and clinicopathological parameters. **a**: TNM stage; **b**: distant metastasis; **c**: lymph node metastasis; **d**: tumor invasion depth

Twelve studies reported the connection between SNHG6 expression and TNM stage (III/IV vs I/II) in 848 patients. Since of the samples were not heterogeneous (I2 = 0.0%, *p* = 0.68), the fixed-effect model was applied to calculate the accumulated OR and its 95% CI. The results indicated that patients with increased SNHG6 expression were prone to advanced TNM stage (OR = 3.31, 95% CI = 2.46–4.45, *P* < 0.001, Table 2, Fig. 2a).

**Table 2:**
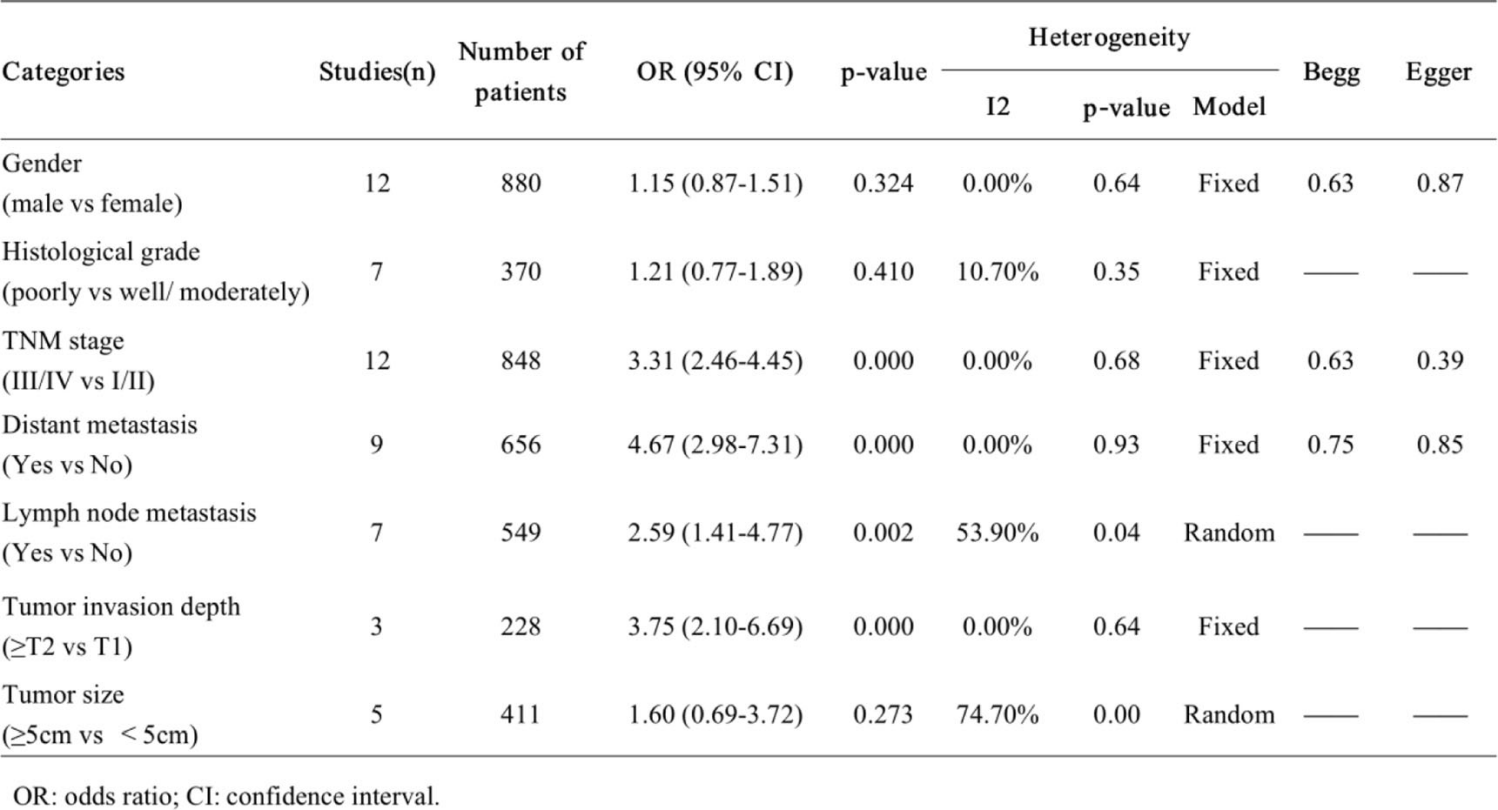
Meta analysis results for the association of over-expressed SNHG6 with clinicopathological parameters

*OR* odds ratio, *CI* confidence interval

#### SNHG6 and distant metastasis

According to the different expression levels of SNHG6, nine studies revealed 656 patients with distant metastasis (yes vs no). We adopted the fixed-effect framework because no heterogeneity was observed (I2 = 0.0%, *p* = 0.93). The pooled results suggested that distant metastasis tended to occur in oncological patients with a high SNHG6 expression level (OR = 4.67, 95% CI = 2.98–7.31, P < 0.001, Table 2, Fig. 2b).

#### SNHG6 and lymph node metastasis

Seven studies that included 549 patients presented data about lymph node metastasis (yes vs no) based on the various expression levels of SNHG6. With obvious heterogeneity among the included studies (I2 = 53.9%, *p* = 0.04), the random-effect model was adopted to generate the cumulative OR, together with the corresponding 95% CI. Our results demonstrated that cancer patients with elevated SNHG6 expression had a higher incidence of lymph node metastasis (OR = 2.59, 95% CI = 1.41–4.77, *P* = 0.002, Table 2, Fig. 2c).

#### SNHG6 and tumor invasion depth

Three studies involving 228 patients elucidated the link between SNHG6 expression and tumor invasion depth (≥T2 vs T1). There was no heterogeneity detected across the research studies (I2 = 0.0%, *p* = 0.64); consequently, a fixed-effect model was performed to calculate the pooled OR and the corresponding 95% CI, which showed statistical significance (OR = 3.75, 95% CI = 2.10–6.69, *P* < 0.001, Table 2, Fig. 2d), suggesting that cancer patients with upregulated SNHG6 expression suffer from deeper tumor invasion.

Additionally, we conducted a meta-analysis to evaluate the association between SNHG6 expression and other clinical characteristics in cancer patients (Table 2). Nevertheless, overexpression of SNHG6 had no significant relation to sex (male vs female) (OR = 1.15, 95% CI = 0.87–1.51, *P* = 0.324, fixed-effect model), histological grade (poorly/others vs well/moderately) (OR = 1.21, 95% CI = 0.77–1.89, *P* = 0.410, fixed-effect model) and tumor size (≥5 cm vs < 5 cm) (OR = 1.60, 95% CI = 0.69–3.72, *P* = 0.273, random-effect model).

### The correlation between SNHG6 expression and the prognosis of human cancers

**Fig. 3:**
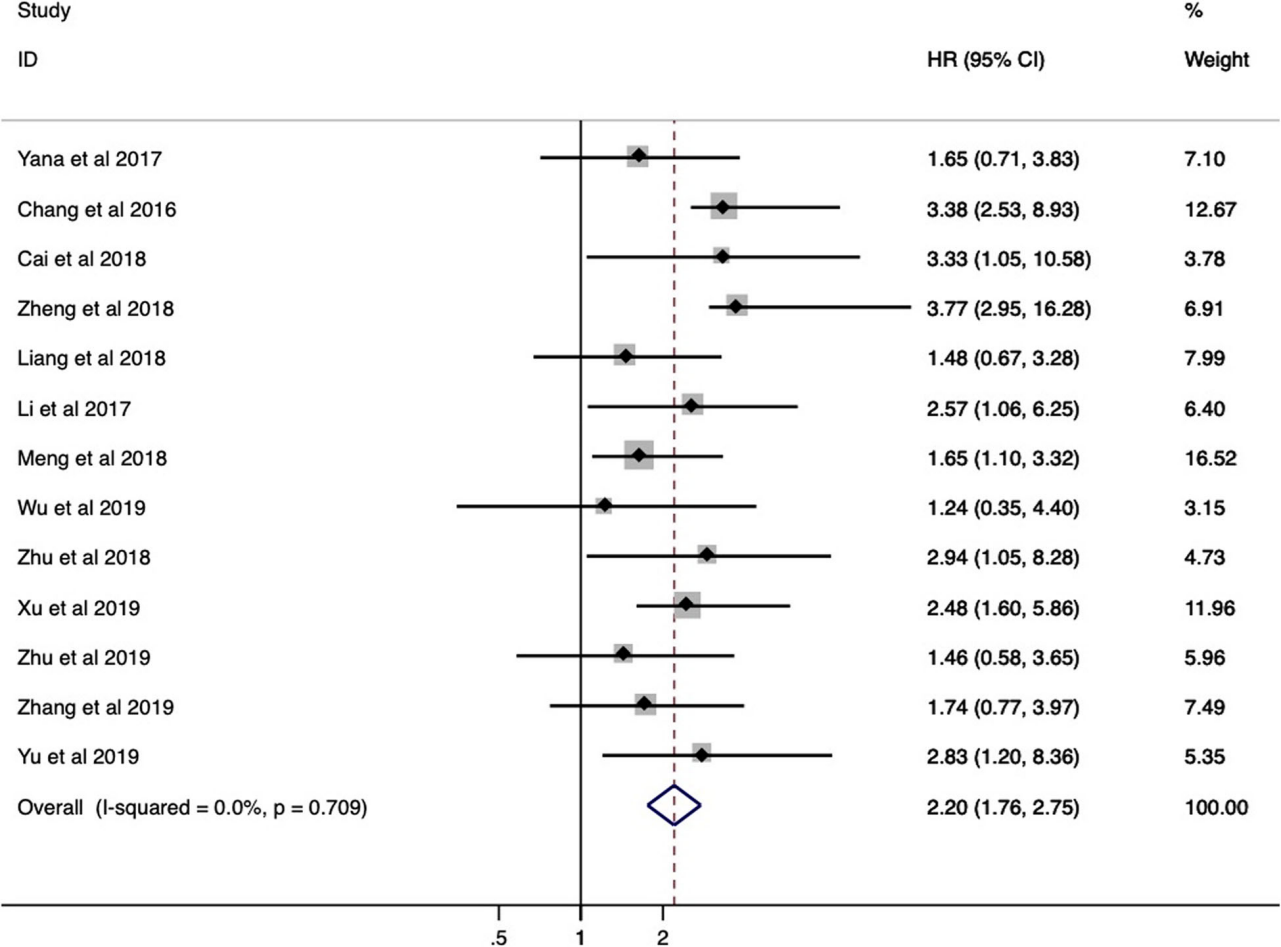
Forest plot for the association between SNHG6 expression and OS

**Fig. 4:**
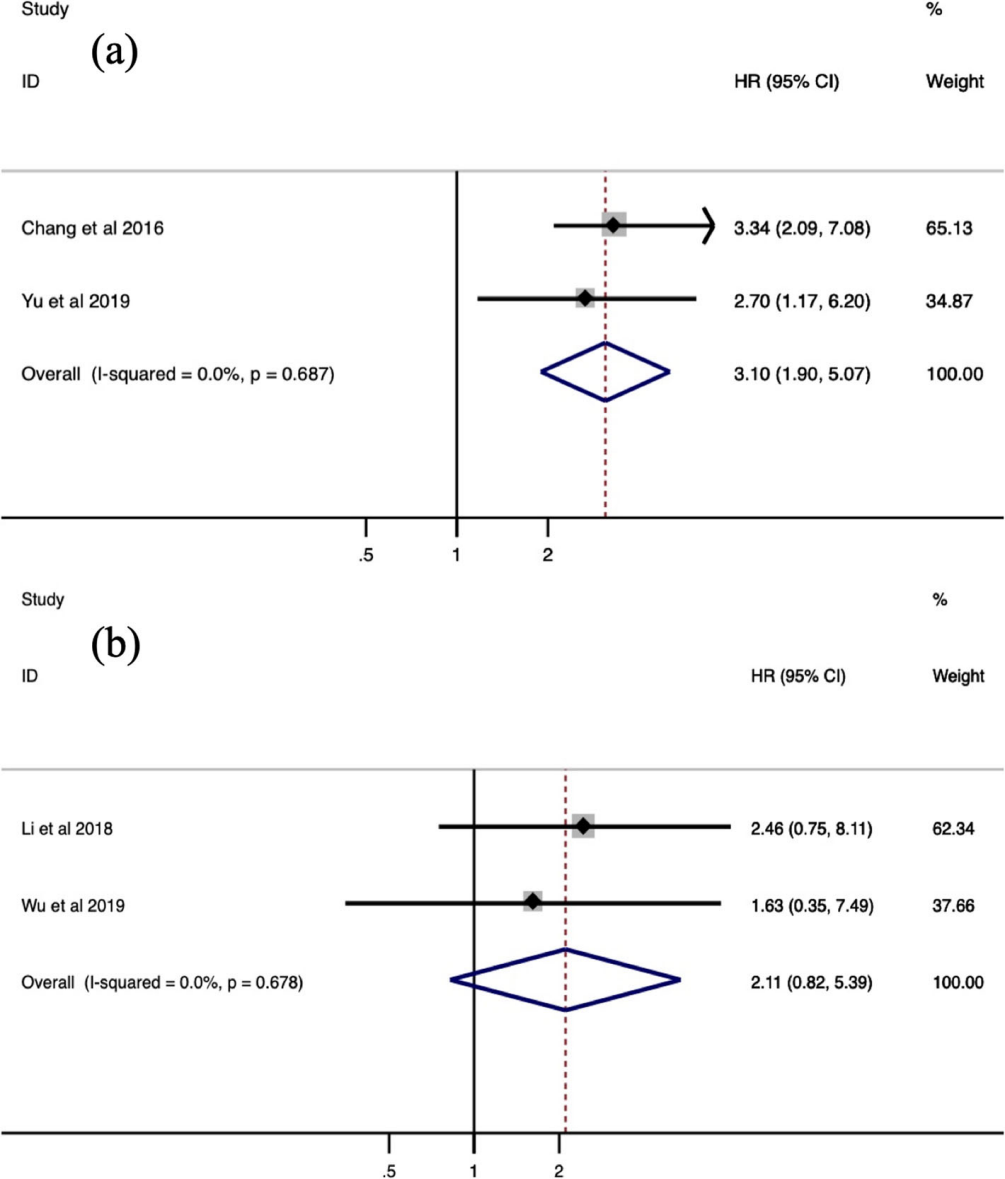
Forest plot for the association between SNHG6 expression and RFS (**a**) with PFS (**b**)

To further understand the prognostic utility of SNHG6, we investigated the correlation of augmented SNHG6 expression and primary survival endpoint (OS/RFS/PFS). As shown in Fig. 3 to Fig. 4, heterogeneity was nonexistent between studies for OS (I2 = 0.0%, *p* = 0.709), RFS (I2 = 0.0%, *p* = 0.687) and PFS (I2 = 0.0%, *p* = 0.678), so fixed-effect models were used. The collective results showed that positive expression of SNHG6 appeared to be significantly correlated with poor OS (HR = 2.20, 95% CI = 1.76–2.75, *P* < 0.001) and RFS (HR = 3.10, 95% CI = 1.90–5.07, *P* < 0.001) in patients with human cancers, while no connection was identified between SNHG6 expression and PFS (HR = 2.11, 95% CI = 0.82–5.39, *P* = 0.120). In other words, SNHG6 expression merits consideration as a prognostic biomarker of OS and RFS. Considering the small number of available studies for RFS and PFS, subgroup analysis, publication bias and sensitivity analysis were only performed for OS.

### Subgroup analysis

Next, we proceeded with subgroup analyses stratified by sample size, tumor type, and extracted method for OS. As illustrated in Table 3, the combined HRs for large and small sample sizes were 2.58 (95% CI = 1.51–4.43, *P* = 0.001) and 2.12 (95% CI = 1.66–2.72, *P* < 0.001) when using 100 patients as the threshold. In addition, we found that high SNHG6 expression was a powerful prognostic marker for shorter OS in patients with digestive system cancer (HR = 2.49, 95% CI = 1.84–3.36, P < 0.001), glioma (HR = 1.88, 95% CI = 1.14–3.09, *P* = 0.013) and osteosarcoma (HR = 2.43, 95% CI = 1.30–4.54, *P* = 0.005), but it was not indicative of other cancers (HR = 1.41, 95% CI = 0.72–2.76, *P* = 0.319). For the extracted method of effect size, the summary HRs were 2.52 for the direct extraction group (95% CI = 1.89–3.37, P < 0.001) and 1.79 for the indirect extraction group (95% CI = 1.26–2.55, *P* = 0.001).

**Table 3:**
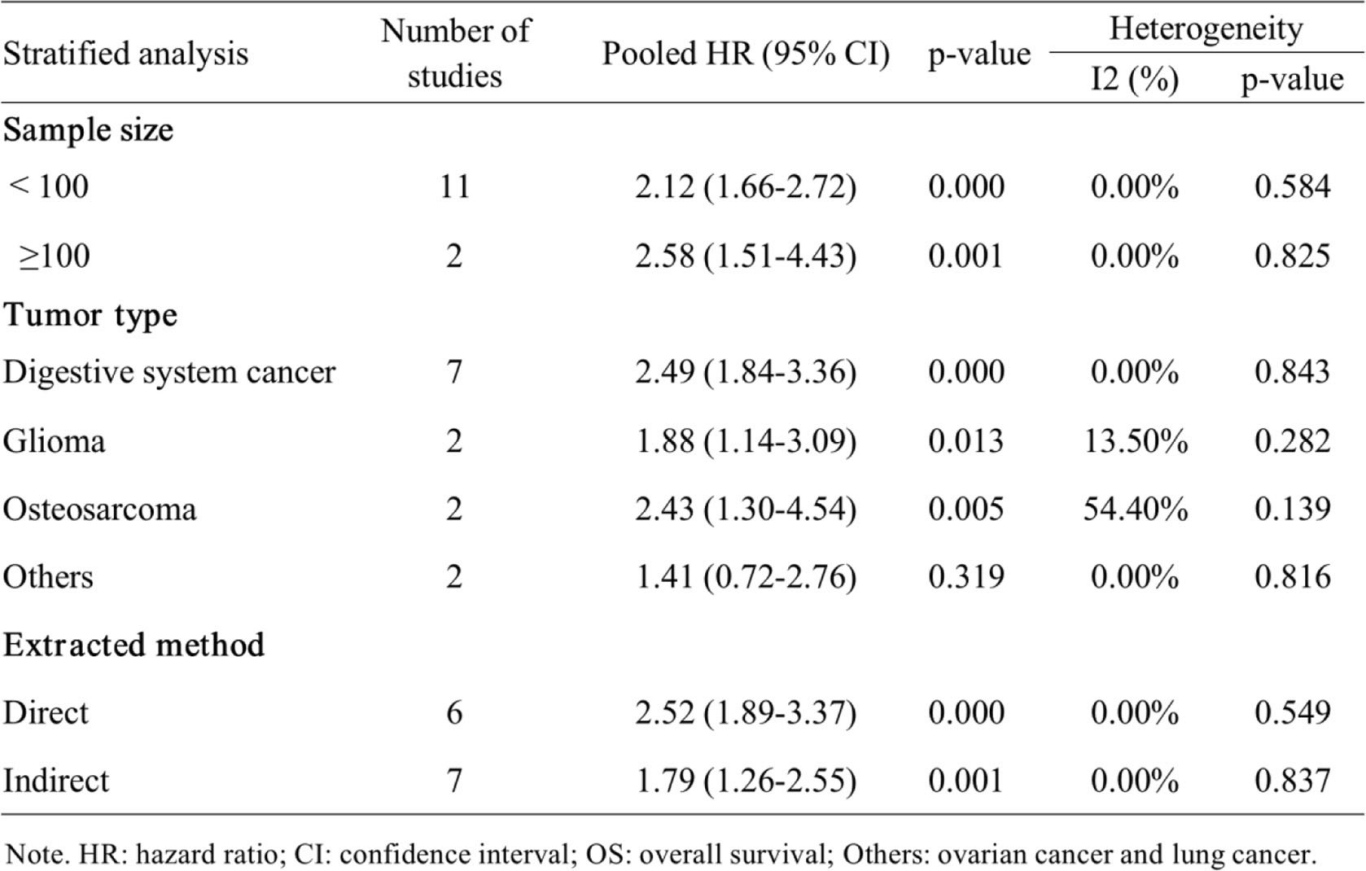
The results of subgroup analyses for OS

*HR* hazard ratio, *CI* confidence interval, *OS* overall survival; Others: ovarian cancer and lung cancer

### Publication bias

**Fig. 5:**
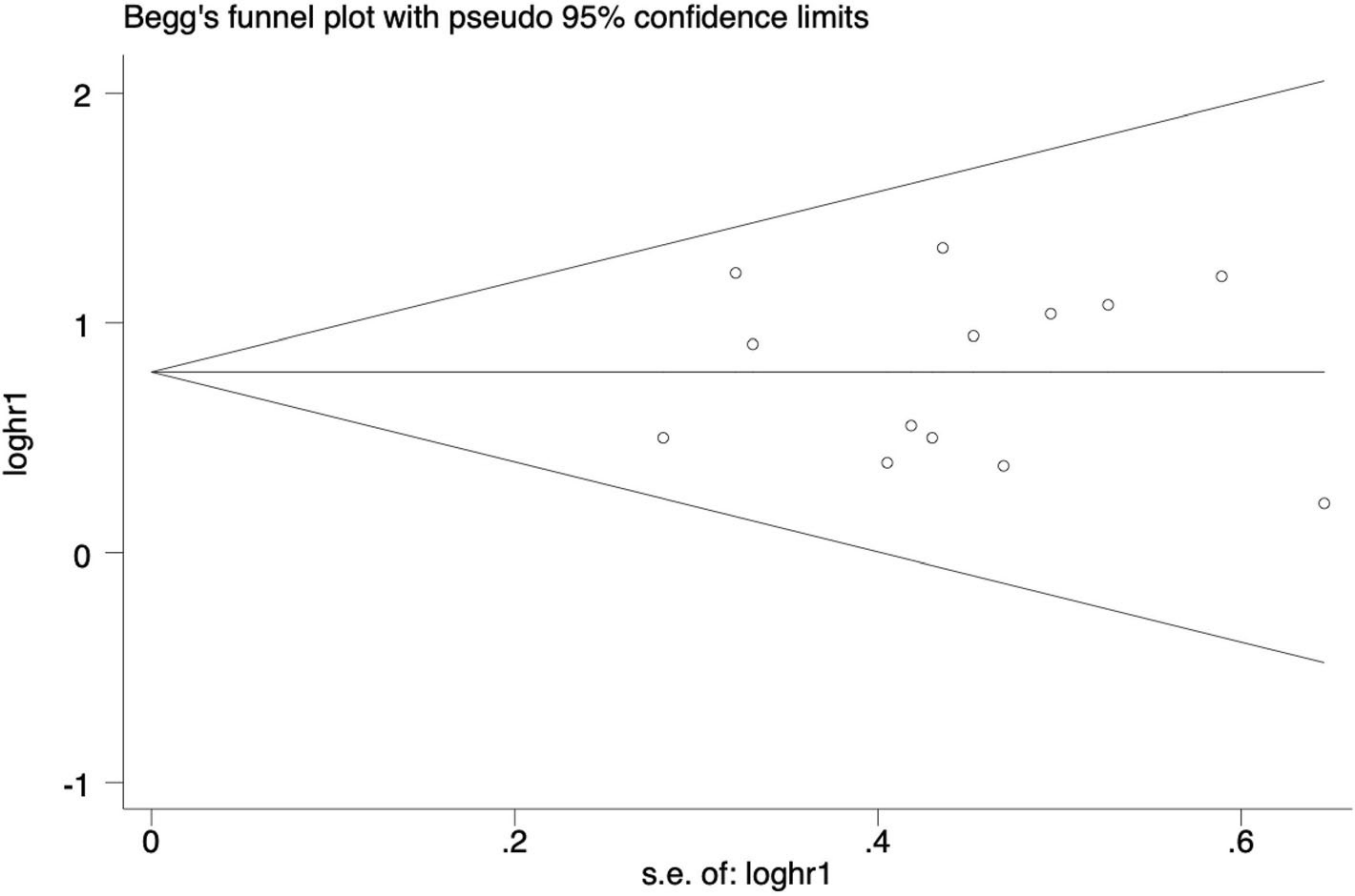
Funnel plot analysis of potential publication bias for OS

Both qualitative and quantitative methods were employed to examine the possible publication bias regarding the outcome of OS. It was evident that the points distributed on the funnel plot were practically symmetrical (Fig. 5). Consistently, when checked by Begg’s (*P* = 0.583) and Egger’s test (*P* = 0.920), the results displayed that no apparent publication bias was found in this meta-analysis. Furthermore, we also examined the publication bias with respect to sex, TNM stage or distant metastasis and drew a similar conclusion (Table 2). Begg’s and Egger’s test for other clinical parameters were not applicable owing to the limited number of eligible publications.

### Sensitivity analysis

**Fig. 6:**
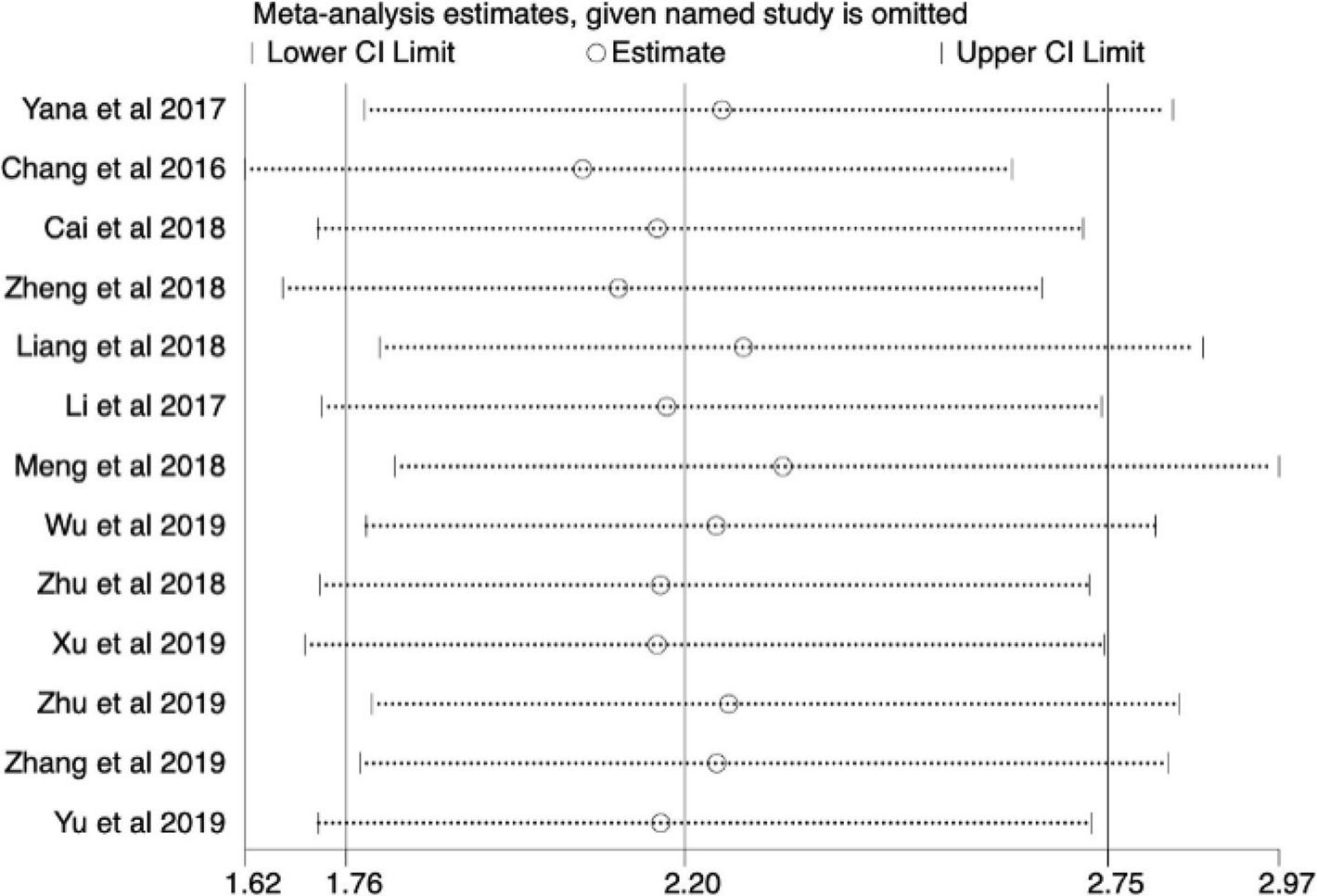
Sensitivity analysis of the relationship between SNHG6 expression and OS

For the purpose of assessing the reliability and stability of our results, sensitivity analysis was contacted by sequentially omitting any individual cohort analysis. Fortunately, the pooled HR for OS was not influenced, which meant increased credibility (Fig. 6).

## Discussion

The formation of tumors is a multifactorial and multi-step process in which lncRNAs act as regulators engaging in gene silencing and activation at epigenetic, transcriptional and post-transcriptional levels [29]. Evidence supports that deregulation of lncRNAs may contribute to various malignant biological behaviours by exerting tumor-promoting or -suppressing functions. With the deepening of research, a wide range of cellular processes, such as alternative splicing control and translation, chemical RNA modification, pre-RNA processing and host mRNA stabilisation, have been demonstrated to be related to the dysregulation of lncRNAs [30, 31]. Additionally, the interaction network of mRNAs and lncRNAs plays vital roles in the occurrence and recurrence of diverse cancers, and the regulatory mechanism may be linked to the competition in binding miRNA targets [32, 33].

SNHG6, as a novel cancer-related lncRNA, is located at chromosome 8q13 and has an evident connection with the structural integrity of the translation initiation complex and ribosomes, and it has become a new frontier of research [34]. SNHG6 was first uncovered to be involved in the regulation of hepatocellular carcinoma. Mechanistically, SNHG6 was identified to abolish miRNA-induced repression of ZEB1 by binding miR-101-3p and inducing epithelial-mesenchymal transition (EMT), thus modulating tumor growth and metastasis [22]. Chen et al. also reported that SNHG6 functioned as a competing endogenous RNA (ceRNA) for miR-181a-5p to regulate E2F5 expression, thereby leading to arrested cell cycle, suppressed cell proliferation, and inhibited cell migration in the development of colorectal cancer [14]. In addition, the underlying mechanisms of SNHG6 have been extensively elucidated in other different types of cancer via numerous molecular pathways, including targeting of TAK1/JNK and Wnt/β-catenin signalling pathway-relevant genes [35], inhibition of the expression of MAPK6 by upregulating miR-26a-5p [36], and so on. Collectively, the above findings further supported our hypothesis that SNHG6 was a potential independent factor involved in tumorigenesis and tumor progression.

In the current meta-analysis, we explored the possibility of a relationship between SNHG6 expression levels and cancer prognostic parameters and pathological attributes by incorporating 14 studies. It was demonstrated that patients with high expression of SNHG6 exhibited an increased risk of unfavourable OS and RFS compared with that in patients with low SNHG6 expression. To assess the application of the above analysis for individual cancers, subgroup analysis was carried out, which showed similar outcomes regardless of the alterations in sample size, tumor type, and extraction method for most cancers. The reason why ovarian cancer and lung cancer analyses had *p*-values greater than 0.05 might be ascribed to limited number of studies and discrepancies in the definitions of cut-off values, measurements, and experimental procedures. Simultaneously, we found that patients with high levels of SNHG6 in cancer tissues had a tendency to develop advanced TNM stage, earlier distant metastasis, positive lymph node metastasis, and deeper tumor invasion. The possible relationship between SNHG6 expression and tumor size and histological grade merits further exploration. Moreover, there was no conspicuous heterogeneity and publication bias of OS and clinical features throughout the study, providing strong evidence of the authenticity of our results. Furthermore, sensitivity analyses were also performed to verify the robustness of the results by the removal of a single study in sequence. Taken together, it was found that SNHG6 is promising candidate for predicting medical outcomes across various types of tumors.

Nevertheless, it is worth noting that there are several limitations that should be mentioned here. First, taking publication bias into consideration, all included subjects originated from China, which led to our results not being representative of western countries to some extent. Second, the type of cancers and total sample size were comparatively smaller, reducing the accuracy of our findings. Moreover, selection bias may exist in light of language restrictions, which could favour the published articles with positive data in English. Third, HRs were obtained from a large proportion of the clinical literature by reconstructing survival curves, so the clinical value of SNHG6 may be inevitably exaggerated. Fourth, it should be taken into account that variation in therapeutic regimens and cut-off definitions might result in an impact on survival outcomes and an overestimate of the prognostic significance of SNHG6 in human cancers.

## Conclusions

Collectively, our meta-analysis preliminarily suggests that SNHG6 could serve as a potential biomarker for predicting prognosis and clinical features in patients with multiple types of cancer. However, multicentre, large-scale, and high-quality studies with normalization are needed to confirm our results. Additionally, the subsequent application of SNHG6 as a prognostic indicator in the routine clinical guidance of cancers deserves further exploration.

## Data Availability

All data are included in this article.

## Abbreviations

ceRNA: Competing endogenous RNA
CIs: Confidence intervals
EMT: Epithelial-mesenchymal transition
HRs: Hazard ratios
ISH: In situ hybridization
lncRNAs: Long non-coding RNAs
ncRNAs: Non-coding RNAs
NOS: Newcastle-Ottawa Scale
ORFs: Open-reading frames
ORs: Odds ratios
OS: Overall survival
PFS: Progression-free survival
qRT-PCR: Quantitative reverse transcription-polymerase chain reaction
RFS: Disease-free survival
SNHG6: Small nucleolar RNA host gene 6
